# Assembly of Zn^II^ and Cd^II^ coordination polymers with different dimensionalities based on the semi-flexible 3-(1*H*-benzimidazol-2-yl)propanoic acid ligand

**DOI:** 10.1107/S2056989017017534

**Published:** 2018-01-01

**Authors:** Xiao-Yan Li, Yong-Qiong Peng, Juan Li, Wei-Wei Fu, Yang Liu, Yu-Ming Li

**Affiliations:** a Key Laboratory of Functional Organometallic Materials of General Colleges and Universities in Hunan Province, College of Chemistry and Materials Science, Hengyang Normal University, Hengyang 421008, People’s Republic of China

**Keywords:** crystal structure, two-dimensional coordination polymers, Zn^II^, one-dimensional coordination polymers, Cd^II^, MOFs

## Abstract

Two new coordination polymers, namely, poly[[μ_3_-3-(1*H*-benzimidazol-2-yl)propionato]zinc(II)], [Zn(C_10_H_8_N_2_O_2_)]_*n*_, (**1**), and poly[bis­[μ_2_-3-(1*H*-benzimid­azol-2-yl)propionato]cadmium(II)], [Cd(C_10_H_8_N_2_O_2_)_2_]_*n*_, (**2**) have been synthesized from 3-(1*H*-benzoimidazol-2-yl)propanoic acid ligands through a mixed-ligand synthetic strategy under a solvothermal environment, and studied by single-crystal X-ray diffraction. Complex **1** crystallizes in the ortho­rhom­bic space group *Pbca* and features a two-dimensional structure formed by a binuclear Zn_2_O_4_ core. Complex **2**, however, crystallizes in the monoclinic space group *P*2_1_/*c* and forms a one-dimensional chain structure. The Zn^II^ and Cd^II^ ions have different coordination numbers and the 3-(1*H*-benzoimidazol-2-yl)propano­ate ligands display different coordination modes. The structures reported here show the importance of the selection of metal ions and suitable ligands.

## Abstract 2

Two unprecedented two-dimensional and one-dimensional Zn^II^ and Cd^II^ complexes based on a semi-flexible ligand have been structurally identified.

## Chemical context   

The structures of coordination polymers are strongly influenced by the organic ligands and metal ions and it is important to choose suitable ligands and metal ions under appropriate synthetic conditions to synthesize coordination complexes with inter­esting structures. The exploration of metal–organic frameworks (MOFs) have received much attention because of their intriguing architectures and wide range of potential applications in different fields (Castellanos *et al.*, 2016 ▸; Zhang *et al.*, 2016 ▸; Kumar *et al.*, 2015 ▸; Liu *et al.*, 2016 ▸; Müller-Buschbaum *et al.*, 2015 ▸; Duerinck & Denayer, 2015 ▸; Mohan *et al.*, 2015 ▸). The assembly of Zn^II^ (Jurcic *et al.*, 2015 ▸; Karmakar *et al.*, 2016*a* ▸,*b* ▸; Liang *et al.*, 2016 ▸; Wannapaiboon *et al.*, 2015 ▸; Ying *et al.*, 2015 ▸) and Cd^II^ (Xiao *et al.*, 2015 ▸, Wu *et al.*, 2011 ▸, Hu *et al.*, 2015 ▸, Cao *et al.*, 2014 ▸, Zhang *et al.*, 2015 ▸) ions with multidentate nitro­gen-containing ligands has produced various MOFs with fascinating structures and luminescent properties. The selection of chelating or bridging organic linkers often favors a structure-specific assembly and the factors that govern the formation of such complexes are complicated and include not only the nature of the Zn^II^ and Cd^II^ ions and ligand structure but also anion-directed inter­actions as well as reaction conditions. In order to explore the coordination chemistry of this type of ligand, 3-(1*H*-benz­imid­azol-2-yl) propanoic acid (H_2_BIP) was chosen in the present study to construct new coordination polymers. A two-dimensional Zn^II^ polymer and a one-dimensional Cd^II^ coord­ination polymer have been obtained.
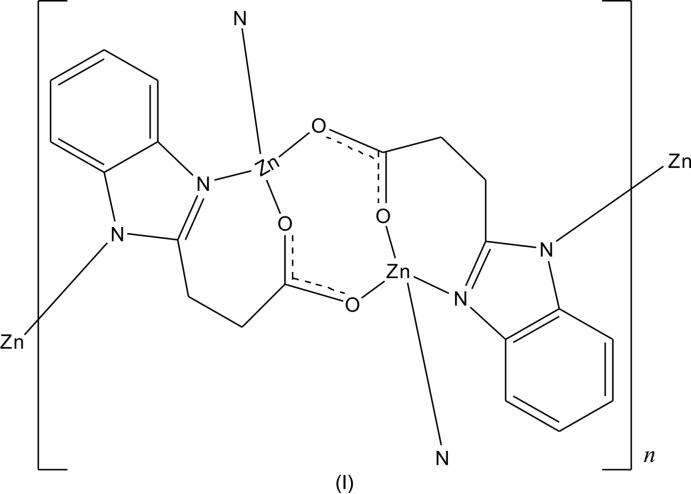
 
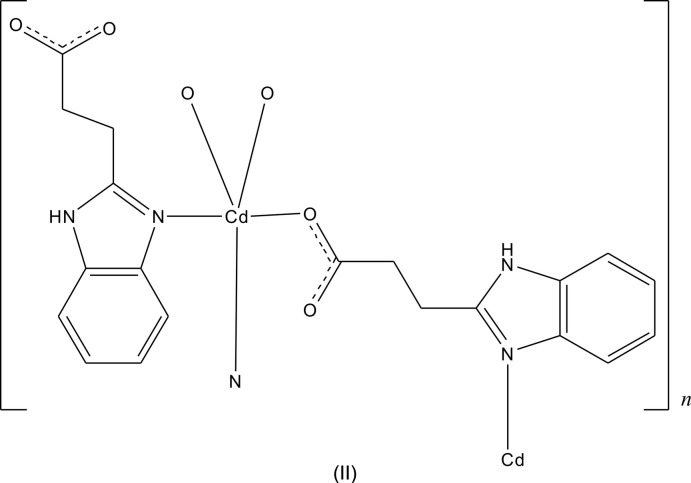


## Structural commentary   

**Figure 1:**
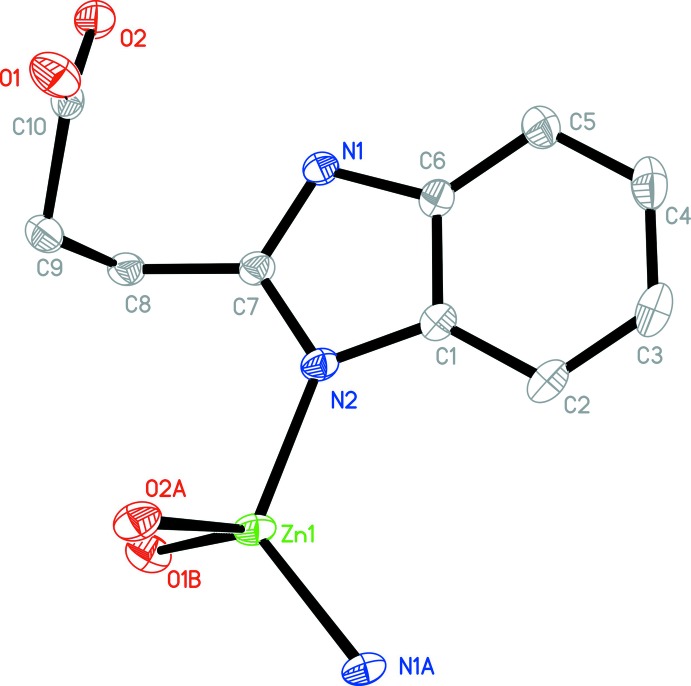
The asymmetric unit of **1**, with additional symmetry-related atoms. The displacement ellipsoids are drawn at the 30% probability level [symmetry codes: (A) −*x* + 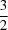, *y* + 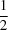, *z*; (B) *x* − 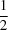, −*y* + 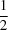, −*z*)].

**Figure 2:**
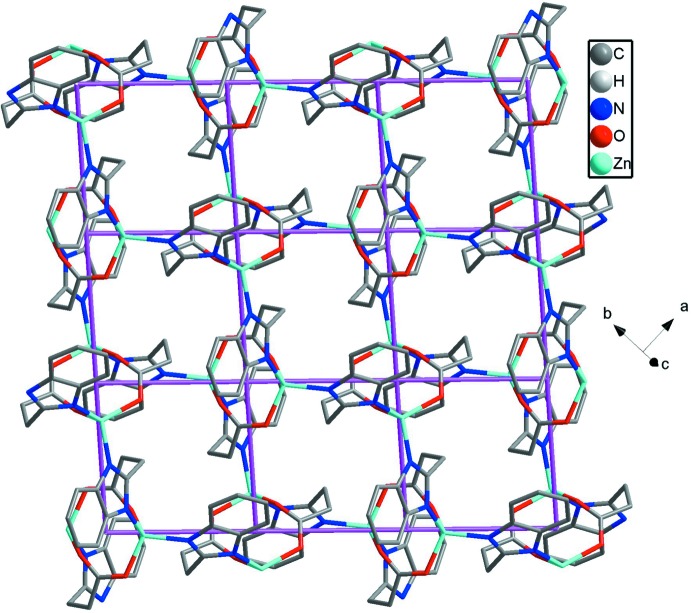
A perspective view of the 4-connected nodes in **1**.

**Figure 3:**
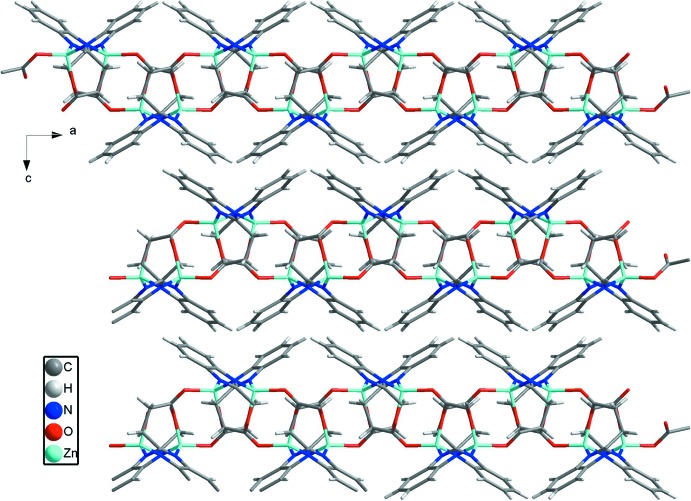
View of the three-dimensional framework of **1** formed by two-dimensional undulating sheets and van der Waals forces.

Complex **1** crystallizes in the ortho­rhom­bic crystal system in the centrosymmetric space group *Pbca*. The 3-(1*H*-benzoimdazol-2-yl)propano­nic acid ligand deprotonates completely when bonding to Zn^II^ ions. The asymmetric unit of **1** consists of one Zn^II^ ion and one 3-(1λ^2^-benzoimidazol-2-yl)propano­ate anion. Geometric parameters are given in Table 1 ▸. As shown in Fig. 1 ▸, the Zn^II^ ion has a tetra­hedral ZnO_2_N_2_ environment completed by N2 from one 3-(1λ^2^-benzoimid­azol-2-yl)propano­ate anion, O2(−*x* + 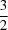, *y* + 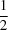, *z*) and N1(−*x* + 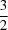, *y* + 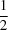, *z*) from the second 3-(1λ^2^-benzoimidazol-2-yl)propano­ate anion and O1(*x* − 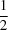, −*y* + 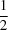, −*z*) from the third 3-(1λ^2^-benzoimidazol-2-yl)propano­ate anion. All the Zn—N/O bond distances [Zn—O: 1.9563 (16)–2.0208 (17) and Zn—N: 1.9624 (18)–1.9661 (16) Å] and the bond angles around Zn1 [99.22 (6)–120.28 (7)°] fall into the normal range. Each 3-(1λ^2^-benzoimidazol-2-yl)propano­ate anion shows a tridentate chelating mode bridging three Zn^II^ ions with the Zn⋯Zn distances of 4.066 (1), 5.870 (2) and 6.965 (2) Å. Zn1 and the symmetry-related Zn1 forming the shortest distance are bridged by O1 and O2 to form a binuclear Zn_2_ cluster. Adjacent clusters are connected by a Zn—N bond of 1.9661 (16) Å to generate 2D square-grid (4,4) layers (Fig. 2 ▸). As there are no classical hydrogen bonds in **1**, these layers are packed by normal van der Waals forces into an extended 3D framework (Fig. 3 ▸).

**Figure 4:**
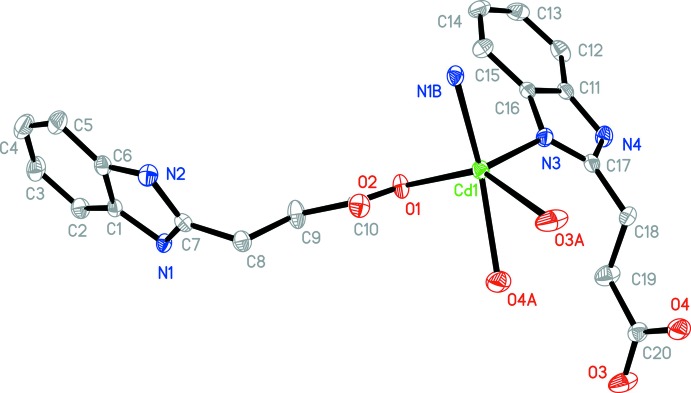
The asymmetric unit of **2**, with additional symmetry-related atoms. The displacement ellipsoids are drawn at the 30% probability level [symmetry codes: (A) −*x*, −*y*, −*z* + 1; (B) *x*, *y* − 1, *z*].

**Figure 5:**
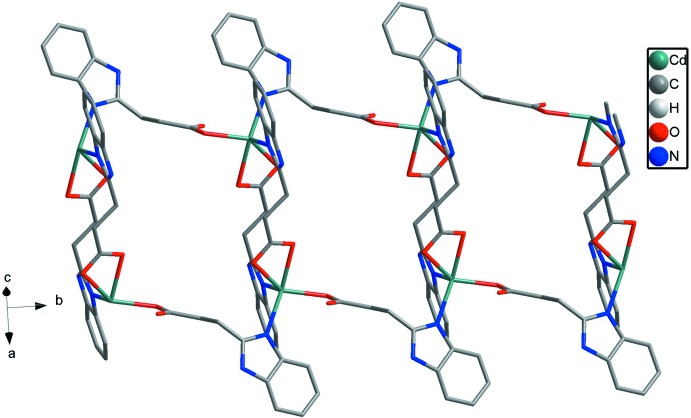
A view of the one-dimensional ladders in **2**.

**Figure 6:**
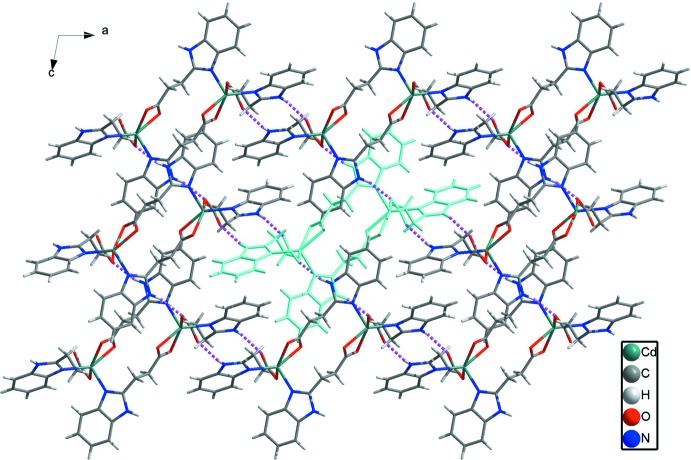
A perspective view of the three-dimensional frameworks in **2** formed by one-dimensional ladders and N—H⋯O hydrogen bonds (Table 3 ▸). The hydrogen bonds are shown as dashed lines.

Complex **2** crystallizes in the monoclinic crystal system in the centrosymmetic space group *P*2_1_/*c*. The 3-(1*H*-benzo­imid­azol-2-yl)propano­nic acid ligands do not deprotonate completely when bonding to Cd^II^ ions. Geometric parameters are given in Table 2 ▸. As shown in Fig. 4 ▸, the Cd^II^ ion is five-coordinated by N3 from one 3-(1*H*-benzoimidazol-2-yl)propano­ate anion, N1(*x*, *y* − 1, *z*) from the second 3-(1*H*-benzoimidazol-2-yl)propano­ate anion, O1 from the third and O3(−*x*, −*y*, −*z* + 1) and O4(−*x*, −*y*, −*z* + 1) from the fourth. All the Cd—N/O bond distances [Cd—O: 2.285 (2)–2.362 (2) and Cd—N: 2.262 (3)–2.271 (3) Å] and the bond angles around Cd1 [55.44 (9)–146.52 (9)°] fall into the normal range. A distance of 2.667 (2) Å between Cd1 and O2 indicates the existence of a weak inter­action between them. Two HBIP^−^ anions connects two Cd^II^ ions with one bidentate carboxyl­ate and one N atom forming end-to-end binuclear Cd_2_ cluster with a distance of 7.274 (1) Å. The other two HBIP^−^ anions act as bridges to join two neighboring binuclear Cd_2_ clusters with one monodentate carboxyl­ate and one N atom to generate 1D ladders along the *b*-axis direction (Fig. 5 ▸). In the crystal, N—H⋯O hydrogen bonds (Table 3 ▸) and π–π inter­actions involv­ing the imidazole rings and benzimidazole ring systems with centroid–centroid distances of 3.569 (2) and 3.838 (2) Å connect the 1D ladders along *a*- and *c*-axis directions into an extended 3D framework (Fig. 6 ▸). Although there are large potential voids within the 1D ladders (7.274 × 8. 025 Å based on the distances of the Cd^II^ ions), they are inter­blocked by adjacent ladders.

## Supra­molecular features   

The structures and the coordination modes of complexes **1** and **2** are quite different, which may be ascribed to a diverse metal coordination habit. The crystal structure of a Zn^II^ complex based on H_2_BIP is reported for the first time. In a comparison with its counterparts based on similar benzo­imidazole carb­oxy­lic acids ligands, benzimidazole-2-butanoic acid (H_2_BIB) and 2-(1*H*-benzimidazol-2-yl­thio)­acetic acid (H_2_BITA), the same coord­ination modes are found for **1** (μ_3_-*κN*,*O*: *κO*’: k*N*′ mode, μ_3_-BIP^2−^) and [Zn(BIB)]_*n*_ (μ_3_-*κN*,*O*: *κO*’: k*N*′ mode, μ_3_-BIB^2−^; Zhang *et al.*, 2015 ▸) and different coordination modes are found for **1** and [Zn_2_(HBITA)_4_]·(DMF)_2_·(H_2_O)_2_ (μ_2_-*κN*: *κO* mode, μ_2_-HBITA^−^ and μ_1_-*κN*,*O* mode, μ_1_-HBITA^−^; Yu *et al.*, 2010 ▸), [Zn_2_(HBITA)_4_]_*n*_ (μ_2_-*κN*: *κO* mode, μ_2_-HBITA^−^; Yu *et al.*, 2010 ▸). Different dimensionalities, like 2D for **1**, 3D for [Zn(BIB)]_*n*_, 0D for [Zn_2_(HBITA)_4_]·(DMF)_2_·(H_2_O)_2_ and 2D for [Zn_2_(HBITA)_4_]_*n*_ are also found. Cd^II^ complexes based on H_2_BIP have already been observed with the appropriate Et_3_N reagent in a EtOH/H_2_O mixed solvent. By selection of the EtOH/H_2_O mixed solvent without any basic reagent, complex **2** was obtained with a relatively simple coordination mode (μ_2_-*κN*: *κO*,*O*′ mode, μ_2_-HBIP^−^) in comparison with diverse modes in {[Cd_5_Cl_2_(HBIP)_4_(BIP)_2_]·4DMF}_*n*_ (μ_2_-*κN*,*O*: *κO*,*O*′ mode, μ_2_-HBIP^−^, μ_3_-*κN*,*O*: κ*O*,*O*′: *κN*’ mode, μ_3_-BIP^2−^, μ_3_-*κN*,*O*: *κO*,*O*′: *κO*’ mode, μ_3_-HBIP^−^; Zheng *et al.*, 2012 ▸) and [Cd_3_(HBIP)_2_(BIP)_2_]_*n*_ (μ_3_-*κN*,*O*: *κO*,*O*′: *κO*’ mode, μ_3_-BIP^2−^, μ_4_-*κN*,*O*: *κO*: *κO*’: *κO*’ mode, μ_4_-HBIP^−^; Zheng *et al.*, 2012 ▸). In comparison with its counterpart based on similar benzo­imidazole carb­oxy­lic acids, H_2_BIB, the same coordination modes are found for **2** and [Cd(HBIB)_2_]_*n*_·(H_2_O)_*n*_ (μ_2_-*κN*: *κO*,*O*′ mode, μ_2_-HBIB^−^; Zhang *et al.*, 2015 ▸). Different dimensionalities, such as 1D for **2**, 2D for {[Cd_5_Cl_2_(HBIP)_4_(BIP)_2_]·4DMF}_*n*_, 1D for [Cd_3_(HBIP)_2_ (BIP)_2_]_*n*_ and 2D for [Cd(HBIB)_2_]_*n*_·(H_2_O)_*n*_ were also found. The different coord­ination modes and dimensionalities show the important roles of spacer lengths and flexibilities of ligands. The crystal structures reported here and before show that ligands containing both flexible carb­oxy­lic and benzimidazole groups are suitable for the construction of coordination polymers with inter­esting structures, adopting diverse coordination modes. The significant effect of metal ions, spacer length and flexibility of ligands on the structural assemblies of such crystalline materials is critical to the assemblies of MOFs in some particular systems.

## Database Survey   

Complexes with benzimidazole-based carb­oxy­lic acid, for example, 1*H*-benzimidazole-2-carb­oxy­lic acid (Xia *et al.*, 2013 ▸; Qiao *et al.*, 2013 ▸; Małecki & Maroń, 2012 ▸; Machura *et al.*, 2014 ▸; Fernández *et al.*, 2016 ▸) and 3-(1*H*-benzimidazole-2-yl) propanoic acid (Liu *et al.*, 2015 ▸) have been reported. A limited number of coordination polymers constructed from 3-(1*H*-benzimidazol-2-yl) propanoic acid (H_2_BIP) have been reported including [Cd_3_(HBIP)_2_(BIP)_2_]_*n*_ and [Cd_5_Cl_2_(BIP)_4_ (BIP)_2_]_*n*_ (Zheng *et al.*, 2012 ▸). [Cd_3_(HBIP)_2_(BIP)_2_]_*n*_ presents a fascinating one-dimensional structure with helical character, made of four helical chains weaving together in two reverse orientations. [Cd_5_Cl_2_(BIP)_4_(BIP)_2_] exhibits a distinct (4,4) network and infinite penta­nuclear secondary building units.

## Synthesis and crystallization   

3-(1*H*-Benzimidazol-2-yl)propanoic acid (H_2_BIP) was prepared by a literature method (Delval *et al.*, 2008 ▸). Other reagents and solvents used in the reactions were purchased from Aladdin-Chemical and used without purification.

### Preparation of 1   

H_2_BIP (0.02 mmol, 0.038 g) and Zn(NO_3_)_2_·6H_2_O (0.2 mmol, 0.060 g) were dissolved in EtOH/H_2_O (1:1 *v*/*v*, 8 ml) mixed solvent. The mixture was sealed in a closed vessel and heated at 413 K for 72 h; the mixture was then cooled slowly to room temperature at a rate of 2 K h^−1^. Many pale-yellow block-shaped crystals were collected.

### Preparation of 2   

H_2_BIP (0.02 mmol, 0.038 g), Cd(CH_3_COO)_2_·2H_2_O (0.2mmol, 0.053 g) were dissolved in EtOH/H_2_O (1:1 *v*/*v*, 8 ml) mixed solvent. The mixture was sealed in a closed vessel and heated at 413 K for 72 h; the mixture was then cooled slowly to room temperature at a rate of 2 K h^−1^. Many brown prismatic crystals were collected.

### Refinement   

Crystal data, data collection and structure refinement details are summarized in Table 4 ▸. H atoms on N atoms were found in the difference-Fourier map and were refined isotrop­ic­ally while restraining the N—H distances to 0.86 Å. Other H atoms were generated geometrically and were allowed to ride on their parent atoms in the riding-model approximation, with C—H = 0.93 Å, *U*_iso_(H) = 1.2*U*_eq_(C)(aromatic) and C—H = 0.97 Å, *U*_iso_(H) = 1.5*U*_eq_(C) for methyl hydrogen atoms.

## Supplementary Material

Crystal structure: contains datablock(s) 1, 2. DOI: 10.1107/S2056989017017534/lh5857sup1.cif

Structure factors: contains datablock(s) 1. DOI: 10.1107/S2056989017017534/lh58571sup2.hkl

Structure factors: contains datablock(s) 2. DOI: 10.1107/S2056989017017534/lh58572sup3.hkl

CCDC references: 1589668, 1589667

Additional supporting information:  crystallographic information; 3D view; checkCIF report

## Figures and Tables

**Table 1:** Selected geometric parameters (Å, °) for **1** [Chem scheme1]

Zn1—O1^i^	1.9563 (16)	Zn1—N2	1.9661 (16)
Zn1—N1^ii^	1.9624 (18)	Zn1—O2^ii^	2.0208 (17)
			
O1^i^—Zn1—N1^ii^	118.50 (7)	O1^i^—Zn1—O2^ii^	105.15 (6)
O1^i^—Zn1—N2	106.84 (7)	N1^ii^—Zn1—O2^ii^	99.22 (6)
N1^ii^—Zn1—N2	120.28 (7)	N2—Zn1—O2^ii^	104.42 (6)

Symmetry codes: (i) 
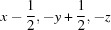
; (ii) 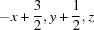.

**Table 2:** Selected geometric parameters (Å, °) for **2** [Chem scheme1]

Cd1—N1^i^	2.262 (3)	Cd1—O3^ii^	2.293 (2)
Cd1—N3	2.271 (3)	Cd1—O4^ii^	2.362 (2)
Cd1—O1	2.285 (2)		
			
N1^i^—Cd1—N3	103.73 (10)	O1—Cd1—O3^ii^	144.01 (9)
N1^i^—Cd1—O1	106.08 (9)	N1^i^—Cd1—O4^ii^	146.52 (9)
N3—Cd1—O1	93.38 (9)	N3—Cd1—O4^ii^	104.51 (10)
N1^i^—Cd1—O3^ii^	100.41 (9)	O1—Cd1—O4^ii^	89.85 (8)
N3—Cd1—O3^ii^	103.63 (10)	O3^ii^—Cd1—O4^ii^	55.44 (9)

Symmetry codes: (i) 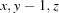; (ii) 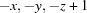.

**Table 3:** Hydrogen-bond geometry (Å, °) for **2** [Chem scheme1]

*D*—H⋯*A*	*D*—H	H⋯*A*	*D*⋯*A*	*D*—H⋯*A*
N2—H2*A*⋯O2^iii^	0.86	2.10	2.823 (4)	141
N4—H4*A*⋯O1^iv^	0.86	2.03	2.862 (4)	161

Symmetry codes: (iii) 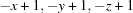; (iv) 
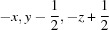
.

**Table 4:** Experimental details

	**1**	**2**
Crystal data		
Chemical formula	[Zn(C_10_H_8_N_2_O_2_)]	[Cd(C_10_H_8_N_2_O_2_)_2_]
*M*_r_	253.55	490.79
Crystal system, space group	Orthorhombic, *P* *b* *c* *a*	Monoclinic, *P*2_1_/*c*
Temperature (K)	296	293
*a*, *b*, *c* (Å)	8.956 (4), 10.697 (5), 20.331 (9)	13.6708 (6), 8.0253 (3), 17.3834 (7)
α, β, γ (°)	90, 90, 90	90, 100.972 (4), 90
*V* (Å^3^)	1947.8 (15)	1872.31 (13)
*Z*	8	4
Radiation type	Mo *K*α	Mo *K*α
μ (mm^−1^)	2.50	1.20
Crystal size (mm)	0.28 × 0.25 × 0.21	0.28 × 0.25 × 0.19

Data collection		
Diffractometer	Bruker SMART CCD area-detector	Bruker SMART CCD area-detector
Absorption correction	Multi-scan (*SADABS*; Bruker, 2012 ▸)	Multi-scan (*SADABS*; Bruker, 2012 ▸)
*T*_min_, *T*_max_	0.541, 0.622	0.923, 1.000
No. of measured, independent and observed [*I* > 2σ(*I*)] reflections	9832, 1725, 1525	6654, 3289, 2685
*R*_int_	0.046	0.029
(sin θ/λ)_max_ (Å^−1^)	0.595	0.595

Refinement		
*R*[*F* ^2^ > 2σ(*F* ^2^)], *wR*(*F* ^2^), *S*	0.024, 0.063, 1.03	0.031, 0.064, 1.06
No. of reflections	1725	3289
No. of parameters	136	262
H-atom treatment	H-atom parameters constrained	H-atom parameters constrained
Δρ_max_, Δρ_min_ (e Å^−3^)	0.29, −0.56	0.33, −0.48

Computer programs: *APEX2* and *SAINT* (Bruker, 2012 ▸), *SHELXL2014* (Sheldrick, 2015 ▸), *OLEX2* (Dolomanov *et al.*, 2009 ▸), *DIAMOND* (Brandenburg, 2008 ▸), *publCIF* (Westrip, 2010 ▸) and *PLATON* (Spek, 2009 ▸).

## supplementary crystallographic information

### Poly[[µ3-3-(1H-benzimidazol-2-yl)propionato]zinc(II)] (1) Crystal data

**Table:**

[Zn(C_10_H_8_N_2_O_2_)]	*D*_x_ = 1.729 Mg m^−^^3^
*M**_r_* = 253.55	Mo *K*α radiation, λ = 0.71073 Å
Orthorhombic, *P**b**c**a*	Cell parameters from 5004 reflections
*a* = 8.956 (4) Å	θ = 3.0–27.6°
*b* = 10.697 (5) Å	µ = 2.50 mm^−^^1^
*c* = 20.331 (9) Å	*T* = 296 K
*V* = 1947.8 (15) Å^3^	Block, yellow
*Z* = 8	0.28 × 0.25 × 0.21 mm
*F*(000) = 1024	

### Poly[[µ3-3-(1H-benzimidazol-2-yl)propionato]zinc(II)] (1) Data collection

**Table:**

Bruker SMART CCD area-detector diffractometer	1525 reflections with *I* > 2σ(*I*)
phi and ω scans	*R*_int_ = 0.046
Absorption correction: multi-scan (SADABS; Bruker, 2012)	θ_max_ = 25.0°, θ_min_ = 2.0°
*T*_min_ = 0.541, *T*_max_ = 0.622	*h* = −10→10
9832 measured reflections	*k* = −12→11
1725 independent reflections	*l* = −24→24

### Poly[[µ3-3-(1H-benzimidazol-2-yl)propionato]zinc(II)] (1) Refinement

**Table:**

Refinement on *F*^2^	Primary atom site location: structure-invariant direct methods
Least-squares matrix: full	Secondary atom site location: difference Fourier map
*R*[*F*^2^ > 2σ(*F*^2^)] = 0.024	Hydrogen site location: inferred from neighbouring sites
*wR*(*F*^2^) = 0.063	H-atom parameters constrained
*S* = 1.03	*w* = 1/[σ^2^(*F*_o_^2^) + (0.0338*P*)^2^ + 0.8648*P*] where *P* = (*F*_o_^2^ + 2*F*_c_^2^)/3
1725 reflections	(Δ/σ)_max_ < 0.001
136 parameters	Δρ_max_ = 0.29 e Å^−^^3^
0 restraints	Δρ_min_ = −0.56 e Å^−^^3^

### Poly[[µ3-3-(1H-benzimidazol-2-yl)propionato]zinc(II)] (1) Special details

**Table:**

Geometry. All esds (except the esd in the dihedral angle between two l.s. planes) are estimated using the full covariance matrix. The cell esds are taken into account individually in the estimation of esds in distances, angles and torsion angles; correlations between esds in cell parameters are only used when they are defined by crystal symmetry. An approximate (isotropic) treatment of cell esds is used for estimating esds involving l.s. planes.

### Poly[[µ3-3-(1H-benzimidazol-2-yl)propionato]zinc(II)] (1) Fractional atomic coordinates and isotropic or equivalent isotropic displacement parameters (Å^2^)

**Table:**

	*x*	*y*	*z*	*U*_iso_*/*U*_eq_	
Zn1	0.61499 (3)	0.44565 (2)	0.08133 (2)	0.02929 (11)	
C1	0.8742 (2)	0.31428 (19)	0.14864 (9)	0.0272 (4)	
C2	0.9402 (3)	0.4128 (2)	0.18261 (10)	0.0361 (5)	
H2	0.8972	0.4919	0.1832	0.043*	
C3	1.0712 (3)	0.3886 (2)	0.21531 (12)	0.0487 (6)	
H3	1.1175	0.4528	0.2384	0.058*	
C4	1.1366 (3)	0.2704 (3)	0.21484 (13)	0.0512 (6)	
H4	1.2268	0.2584	0.2366	0.061*	
C5	1.0711 (3)	0.1710 (2)	0.18305 (11)	0.0402 (5)	
H5	1.1136	0.0917	0.1837	0.048*	
C6	0.9381 (2)	0.19494 (18)	0.14985 (9)	0.0278 (4)	
C7	0.7378 (2)	0.18898 (16)	0.08956 (9)	0.0273 (4)	
C8	0.6243 (2)	0.1407 (2)	0.04189 (11)	0.0326 (5)	
H8A	0.5329	0.1886	0.0464	0.039*	
H8B	0.6018	0.0542	0.0523	0.039*	
C9	0.6791 (2)	0.14918 (19)	−0.02923 (10)	0.0347 (5)	
H9A	0.6001	0.1208	−0.0582	0.042*	
H9B	0.6992	0.2361	−0.0396	0.042*	
C10	0.8184 (2)	0.07312 (18)	−0.04282 (10)	0.0296 (4)	
N1	0.84880 (17)	0.11670 (14)	0.11236 (9)	0.0289 (4)	
N2	0.74575 (17)	0.30823 (13)	0.10974 (8)	0.0268 (4)	
O1	0.91054 (16)	0.11866 (15)	−0.08295 (7)	0.0373 (4)	
O2	0.83644 (16)	−0.03094 (12)	−0.01494 (7)	0.0332 (3)	

### Poly[[µ3-3-(1H-benzimidazol-2-yl)propionato]zinc(II)] (1) Atomic displacement parameters (Å^2^)

**Table:**

	*U*^11^	*U*^22^	*U*^33^	*U*^12^	*U*^13^	*U*^23^
Zn1	0.03268 (17)	0.01695 (16)	0.03822 (18)	0.00017 (8)	0.00399 (10)	0.00205 (9)
C1	0.0323 (10)	0.0252 (10)	0.0240 (10)	−0.0034 (8)	0.0028 (8)	0.0001 (8)
C2	0.0459 (12)	0.0284 (11)	0.0341 (12)	−0.0065 (9)	0.0030 (10)	−0.0084 (9)
C3	0.0548 (14)	0.0475 (14)	0.0437 (13)	−0.0116 (12)	−0.0098 (12)	−0.0148 (12)
C4	0.0507 (14)	0.0570 (16)	0.0460 (14)	−0.0032 (12)	−0.0209 (11)	−0.0069 (13)
C5	0.0438 (12)	0.0384 (12)	0.0382 (12)	0.0045 (10)	−0.0119 (10)	−0.0008 (10)
C6	0.0329 (10)	0.0256 (10)	0.0250 (9)	−0.0030 (8)	−0.0006 (8)	0.0009 (8)
C7	0.0290 (10)	0.0207 (10)	0.0323 (10)	−0.0013 (8)	−0.0003 (8)	0.0000 (8)
C8	0.0283 (10)	0.0235 (10)	0.0459 (13)	0.0017 (8)	−0.0070 (9)	−0.0045 (9)
C9	0.0337 (11)	0.0287 (11)	0.0416 (12)	0.0044 (8)	−0.0113 (9)	−0.0002 (9)
C10	0.0326 (11)	0.0226 (10)	0.0336 (11)	−0.0011 (8)	−0.0119 (9)	−0.0037 (8)
N1	0.0332 (9)	0.0177 (8)	0.0360 (9)	0.0000 (7)	−0.0054 (7)	−0.0009 (7)
N2	0.0294 (9)	0.0193 (8)	0.0317 (9)	−0.0009 (7)	0.0013 (7)	−0.0006 (7)
O1	0.0330 (8)	0.0327 (9)	0.0461 (9)	0.0019 (6)	−0.0032 (6)	0.0111 (7)
O2	0.0423 (8)	0.0211 (7)	0.0360 (8)	0.0020 (6)	−0.0053 (6)	0.0013 (6)

### Poly[[µ3-3-(1H-benzimidazol-2-yl)propionato]zinc(II)] (1) Geometric parameters (Å, º)

**Table:**

Zn1—O1^i^	1.9563 (16)	C6—N1	1.386 (3)
Zn1—N1^ii^	1.9624 (18)	C7—N2	1.342 (2)
Zn1—N2	1.9661 (16)	C7—N1	1.342 (2)
Zn1—O2^ii^	2.0208 (17)	C7—C8	1.496 (3)
C1—C2	1.392 (3)	C8—C9	1.530 (3)
C1—N2	1.398 (2)	C8—H8A	0.9700
C1—C6	1.399 (3)	C8—H8B	0.9700
C2—C3	1.374 (3)	C9—C10	1.515 (3)
C2—H2	0.9300	C9—H9A	0.9700
C3—C4	1.393 (4)	C9—H9B	0.9700
C3—H3	0.9300	C10—O1	1.259 (3)
C4—C5	1.375 (3)	C10—O2	1.260 (2)
C4—H4	0.9300	N1—Zn1^iii^	1.9624 (18)
C5—C6	1.393 (3)	O1—Zn1^iv^	1.9563 (16)
C5—H5	0.9300	O2—Zn1^iii^	2.0207 (17)
			
O1^i^—Zn1—N1^ii^	118.50 (7)	N2—C7—C8	124.22 (17)
O1^i^—Zn1—N2	106.84 (7)	N1—C7—C8	121.86 (16)
N1^ii^—Zn1—N2	120.28 (7)	C7—C8—C9	111.96 (17)
O1^i^—Zn1—O2^ii^	105.15 (6)	C7—C8—H8A	109.2
N1^ii^—Zn1—O2^ii^	99.22 (6)	C9—C8—H8A	109.2
N2—Zn1—O2^ii^	104.42 (6)	C7—C8—H8B	109.2
C2—C1—N2	131.70 (19)	C9—C8—H8B	109.2
C2—C1—C6	120.56 (19)	H8A—C8—H8B	107.9
N2—C1—C6	107.74 (16)	C10—C9—C8	113.88 (16)
C3—C2—C1	117.4 (2)	C10—C9—H9A	108.8
C3—C2—H2	121.3	C8—C9—H9A	108.8
C1—C2—H2	121.3	C10—C9—H9B	108.8
C2—C3—C4	121.8 (2)	C8—C9—H9B	108.8
C2—C3—H3	119.1	H9A—C9—H9B	107.7
C4—C3—H3	119.1	O1—C10—O2	123.33 (19)
C5—C4—C3	121.7 (2)	O1—C10—C9	116.77 (18)
C5—C4—H4	119.1	O2—C10—C9	119.89 (19)
C3—C4—H4	119.1	C7—N1—C6	105.65 (16)
C4—C5—C6	116.7 (2)	C7—N1—Zn1^iii^	123.27 (13)
C4—C5—H5	121.6	C6—N1—Zn1^iii^	130.11 (13)
C6—C5—H5	121.6	C7—N2—C1	105.11 (15)
N1—C6—C5	130.48 (19)	C7—N2—Zn1	126.10 (13)
N1—C6—C1	107.75 (17)	C1—N2—Zn1	128.46 (13)
C5—C6—C1	121.75 (18)	C10—O1—Zn1^iv^	117.83 (13)
N2—C7—N1	113.75 (17)	C10—O2—Zn1^iii^	124.96 (13)
			
N2—C1—C2—C3	−178.1 (2)	N2—C7—N1—Zn1^iii^	−170.56 (13)
C6—C1—C2—C3	1.8 (3)	C8—C7—N1—Zn1^iii^	4.9 (3)
C1—C2—C3—C4	0.0 (4)	C5—C6—N1—C7	−177.4 (2)
C2—C3—C4—C5	−1.8 (4)	C1—C6—N1—C7	0.7 (2)
C3—C4—C5—C6	1.6 (4)	C5—C6—N1—Zn1^iii^	−8.6 (3)
C4—C5—C6—N1	178.1 (2)	C1—C6—N1—Zn1^iii^	169.47 (14)
C4—C5—C6—C1	0.2 (3)	N1—C7—N2—C1	0.6 (2)
C2—C1—C6—N1	179.73 (18)	C8—C7—N2—C1	−174.75 (18)
N2—C1—C6—N1	−0.4 (2)	N1—C7—N2—Zn1	174.49 (13)
C2—C1—C6—C5	−2.0 (3)	C8—C7—N2—Zn1	−0.8 (3)
N2—C1—C6—C5	177.93 (18)	C2—C1—N2—C7	179.8 (2)
N2—C7—C8—C9	90.4 (2)	C6—C1—N2—C7	−0.1 (2)
N1—C7—C8—C9	−84.6 (2)	C2—C1—N2—Zn1	6.1 (3)
C7—C8—C9—C10	61.3 (2)	C6—C1—N2—Zn1	−173.81 (13)
C8—C9—C10—O1	−144.39 (18)	O2—C10—O1—Zn1^iv^	−19.8 (3)
C8—C9—C10—O2	36.4 (3)	C9—C10—O1—Zn1^iv^	160.95 (13)
N2—C7—N1—C6	−0.8 (2)	O1—C10—O2—Zn1^iii^	108.7 (2)
C8—C7—N1—C6	174.63 (18)	C9—C10—O2—Zn1^iii^	−72.1 (2)

Symmetry codes: (i) *x*−1/2, −*y*+1/2, −*z*; (ii) −*x*+3/2, *y*+1/2, *z*; (iii) −*x*+3/2, *y*−1/2, *z*; (iv) *x*+1/2, −*y*+1/2, −*z*.

### Poly[bis[µ2-3-(1H-benzimidazol-2-yl)propionato]cadmium(II)] (2) Crystal data

**Table:**

[Cd(C_10_H_8_N_2_O_2_)_2_]	*F*(000) = 984
*M**_r_* = 490.79	*D*_x_ = 1.741 Mg m^−^^3^
Monoclinic, *P*2_1_/*c*	Mo *K*α radiation, λ = 0.71073 Å
*a* = 13.6708 (6) Å	Cell parameters from 2660 reflections
*b* = 8.0253 (3) Å	θ = 3.0–27.4°
*c* = 17.3834 (7) Å	µ = 1.20 mm^−^^1^
β = 100.972 (4)°	*T* = 293 K
*V* = 1872.31 (13) Å^3^	Prism, brown
*Z* = 4	0.28 × 0.25 × 0.19 mm

### Poly[bis[µ2-3-(1H-benzimidazol-2-yl)propionato]cadmium(II)] (2) Data collection

**Table:**

Bruker SMART CCD area-detector diffractometer	2685 reflections with *I* > 2σ(*I*)
Radiation source: fine-focus sealed tube	*R*_int_ = 0.029
phi and ω scans	θ_max_ = 25.0°, θ_min_ = 2.4°
Absorption correction: multi-scan (SADABS; Bruker, 2012)	*h* = −16→15
*T*_min_ = 0.923, *T*_max_ = 1.000	*k* = −9→6
6654 measured reflections	*l* = −20→14
3289 independent reflections	

### Poly[bis[µ2-3-(1H-benzimidazol-2-yl)propionato]cadmium(II)] (2) Refinement

**Table:**

Refinement on *F*^2^	Primary atom site location: structure-invariant direct methods
Least-squares matrix: full	Secondary atom site location: difference Fourier map
*R*[*F*^2^ > 2σ(*F*^2^)] = 0.031	Hydrogen site location: inferred from neighbouring sites
*wR*(*F*^2^) = 0.064	H-atom parameters constrained
*S* = 1.06	*w* = 1/[σ^2^(*F*_o_^2^) + (0.0234*P*)^2^] where *P* = (*F*_o_^2^ + 2*F*_c_^2^)/3
3289 reflections	(Δ/σ)_max_ < 0.001
262 parameters	Δρ_max_ = 0.33 e Å^−^^3^
0 restraints	Δρ_min_ = −0.48 e Å^−^^3^

### Poly[bis[µ2-3-(1H-benzimidazol-2-yl)propionato]cadmium(II)] (2) Special details

**Table:**

Geometry. All esds (except the esd in the dihedral angle between two l.s. planes) are estimated using the full covariance matrix. The cell esds are taken into account individually in the estimation of esds in distances, angles and torsion angles; correlations between esds in cell parameters are only used when they are defined by crystal symmetry. An approximate (isotropic) treatment of cell esds is used for estimating esds involving l.s. planes.

### Poly[bis[µ2-3-(1H-benzimidazol-2-yl)propionato]cadmium(II)] (2) Fractional atomic coordinates and isotropic or equivalent isotropic displacement parameters (Å^2^)

**Table:**

	*x*	*y*	*z*	*U*_iso_*/*U*_eq_	
Cd1	0.22103 (2)	0.06562 (3)	0.41588 (2)	0.02433 (9)	
C1	0.4463 (3)	1.0429 (4)	0.37316 (19)	0.0254 (8)	
C2	0.4459 (3)	1.1926 (4)	0.33279 (19)	0.0343 (9)	
H2	0.3890	1.2587	0.3224	0.041*	
C3	0.5321 (3)	1.2403 (5)	0.3087 (2)	0.0442 (11)	
H3	0.5337	1.3410	0.2825	0.053*	
C4	0.6164 (3)	1.1402 (5)	0.3229 (2)	0.0503 (11)	
H4	0.6734	1.1750	0.3056	0.060*	
C5	0.6179 (3)	0.9902 (5)	0.3622 (2)	0.0444 (10)	
H5	0.6740	0.9224	0.3710	0.053*	
C6	0.5323 (3)	0.9465 (4)	0.3874 (2)	0.0298 (8)	
C7	0.4118 (3)	0.8209 (4)	0.43435 (18)	0.0263 (8)	
C8	0.3603 (3)	0.6815 (4)	0.46664 (19)	0.0305 (8)	
H8A	0.3197	0.7255	0.5020	0.037*	
H8B	0.4094	0.6069	0.4962	0.037*	
C9	0.2949 (3)	0.5857 (4)	0.4010 (2)	0.0404 (10)	
H9A	0.3250	0.5925	0.3548	0.048*	
H9B	0.2308	0.6413	0.3886	0.048*	
C10	0.2768 (3)	0.4031 (4)	0.4167 (2)	0.0261 (8)	
C11	0.0502 (3)	−0.0701 (4)	0.18100 (19)	0.0274 (8)	
C12	0.0484 (3)	−0.0730 (4)	0.1006 (2)	0.0373 (9)	
H12	−0.0082	−0.1041	0.0648	0.045*	
C13	0.1354 (3)	−0.0273 (4)	0.0773 (2)	0.0390 (10)	
H13	0.1371	−0.0271	0.0241	0.047*	
C14	0.2200 (3)	0.0185 (5)	0.1299 (2)	0.0397 (10)	
H14	0.2769	0.0493	0.1113	0.048*	
C15	0.2218 (3)	0.0193 (4)	0.2096 (2)	0.0336 (9)	
H15	0.2790	0.0490	0.2452	0.040*	
C16	0.1354 (3)	−0.0256 (4)	0.23458 (19)	0.0237 (8)	
C17	0.0228 (3)	−0.0925 (4)	0.30217 (19)	0.0272 (8)	
C18	−0.0301 (3)	−0.1297 (4)	0.3673 (2)	0.0333 (9)	
H18A	0.0190	−0.1575	0.4136	0.040*	
H18B	−0.0717	−0.2271	0.3534	0.040*	
C19	−0.0936 (3)	0.0092 (5)	0.3873 (2)	0.0433 (10)	
H19A	−0.1486	0.0260	0.3436	0.052*	
H19B	−0.0544	0.1107	0.3937	0.052*	
C20	−0.1353 (3)	−0.0190 (5)	0.4602 (2)	0.0317 (9)	
O1	0.21406 (17)	0.3287 (3)	0.36487 (13)	0.0316 (6)	
O2	0.32390 (18)	0.3331 (3)	0.47586 (13)	0.0309 (6)	
O3	−0.1888 (2)	0.0912 (3)	0.48158 (15)	0.0504 (8)	
O4	−0.1163 (2)	−0.1496 (3)	0.49775 (14)	0.0448 (7)	
N1	0.3712 (2)	0.9617 (3)	0.40401 (15)	0.0242 (7)	
N2	0.5075 (2)	0.8066 (3)	0.42631 (16)	0.0313 (7)	
H2A	0.5466	0.7247	0.4426	0.038*	
N3	0.1154 (2)	−0.0401 (3)	0.31043 (16)	0.0277 (7)	
N4	−0.0197 (2)	−0.1100 (3)	0.22566 (16)	0.0323 (7)	
H4A	−0.0800	−0.1408	0.2079	0.039*	

### Poly[bis[µ2-3-(1H-benzimidazol-2-yl)propionato]cadmium(II)] (2) Atomic displacement parameters (Å^2^)

**Table:**

	*U*^11^	*U*^22^	*U*^33^	*U*^12^	*U*^13^	*U*^23^
Cd1	0.02465 (15)	0.02141 (14)	0.02708 (15)	0.00049 (12)	0.00527 (10)	0.00198 (12)
C1	0.026 (2)	0.0254 (19)	0.0239 (17)	−0.0039 (16)	0.0022 (15)	−0.0025 (17)
C2	0.037 (2)	0.029 (2)	0.037 (2)	−0.0006 (18)	0.0046 (17)	0.0021 (19)
C3	0.051 (3)	0.040 (2)	0.042 (2)	−0.015 (2)	0.010 (2)	0.009 (2)
C4	0.039 (3)	0.061 (3)	0.054 (3)	−0.016 (2)	0.015 (2)	0.002 (3)
C5	0.026 (2)	0.048 (2)	0.059 (3)	−0.005 (2)	0.005 (2)	−0.005 (2)
C6	0.027 (2)	0.032 (2)	0.0278 (19)	−0.0043 (17)	−0.0002 (16)	−0.0042 (18)
C7	0.029 (2)	0.0229 (18)	0.0250 (18)	0.0007 (16)	0.0010 (15)	−0.0038 (16)
C8	0.035 (2)	0.0219 (18)	0.034 (2)	0.0000 (17)	0.0061 (17)	0.0028 (17)
C9	0.044 (3)	0.0257 (19)	0.045 (2)	−0.0080 (18)	−0.0073 (19)	0.009 (2)
C10	0.022 (2)	0.0234 (18)	0.035 (2)	0.0023 (16)	0.0105 (16)	0.0010 (18)
C11	0.031 (2)	0.0212 (18)	0.0298 (19)	−0.0004 (17)	0.0061 (16)	−0.0025 (17)
C12	0.051 (3)	0.030 (2)	0.027 (2)	0.0007 (19)	−0.0016 (18)	−0.0008 (18)
C13	0.052 (3)	0.040 (2)	0.027 (2)	0.010 (2)	0.0142 (19)	0.0035 (19)
C14	0.037 (2)	0.045 (2)	0.042 (2)	0.005 (2)	0.0196 (19)	0.002 (2)
C15	0.027 (2)	0.040 (2)	0.034 (2)	0.0014 (18)	0.0064 (17)	0.0027 (19)
C16	0.025 (2)	0.0212 (18)	0.0250 (18)	0.0012 (15)	0.0043 (15)	−0.0018 (16)
C17	0.033 (2)	0.0193 (18)	0.0300 (19)	−0.0023 (16)	0.0080 (16)	−0.0010 (16)
C18	0.032 (2)	0.0331 (19)	0.038 (2)	−0.0099 (18)	0.0142 (17)	−0.0029 (19)
C19	0.047 (3)	0.047 (2)	0.039 (2)	0.013 (2)	0.016 (2)	0.015 (2)
C20	0.024 (2)	0.038 (2)	0.033 (2)	−0.0041 (18)	0.0044 (17)	−0.003 (2)
O1	0.0296 (15)	0.0226 (12)	0.0384 (14)	−0.0051 (11)	−0.0042 (11)	0.0023 (12)
O2	0.0314 (15)	0.0244 (13)	0.0347 (13)	0.0038 (11)	0.0008 (11)	0.0087 (12)
O3	0.055 (2)	0.0548 (17)	0.0489 (16)	0.0248 (15)	0.0285 (15)	0.0199 (15)
O4	0.063 (2)	0.0349 (15)	0.0430 (16)	0.0055 (14)	0.0273 (14)	0.0078 (14)
N1	0.0207 (16)	0.0187 (14)	0.0324 (16)	−0.0001 (12)	0.0034 (12)	0.0043 (13)
N2	0.0227 (17)	0.0265 (15)	0.0426 (17)	0.0053 (14)	0.0004 (14)	0.0050 (15)
N3	0.0290 (18)	0.0281 (16)	0.0269 (16)	−0.0065 (14)	0.0073 (13)	−0.0028 (14)
N4	0.0273 (18)	0.0346 (17)	0.0331 (17)	−0.0078 (14)	0.0012 (14)	−0.0014 (16)

### Poly[bis[µ2-3-(1H-benzimidazol-2-yl)propionato]cadmium(II)] (2) Geometric parameters (Å, º)

**Table:**

Cd1—N1^i^	2.262 (3)	C10—O1	1.269 (4)
Cd1—N3	2.271 (3)	C11—N4	1.378 (4)
Cd1—O1	2.285 (2)	C11—C12	1.393 (5)
Cd1—O3^ii^	2.293 (2)	C11—C16	1.393 (4)
Cd1—O4^ii^	2.362 (2)	C12—C13	1.377 (5)
Cd1—O2	2.667 (2)	C12—H12	0.9300
Cd1—C20^ii^	2.667 (4)	C13—C14	1.382 (5)
Cd1—C10	2.813 (3)	C13—H13	0.9300
C1—C6	1.389 (5)	C14—C15	1.382 (5)
C1—C2	1.391 (4)	C14—H14	0.9300
C1—N1	1.406 (4)	C15—C16	1.382 (5)
C2—C3	1.377 (5)	C15—H15	0.9300
C2—H2	0.9300	C16—N3	1.401 (4)
C3—C4	1.389 (5)	C17—N3	1.316 (4)
C3—H3	0.9300	C17—N4	1.354 (4)
C4—C5	1.382 (5)	C17—C18	1.485 (4)
C4—H4	0.9300	C18—C19	1.495 (5)
C5—C6	1.371 (5)	C18—H18A	0.9700
C5—H5	0.9300	C18—H18B	0.9700
C6—N2	1.386 (4)	C19—C20	1.503 (5)
C7—N1	1.323 (4)	C19—H19A	0.9700
C7—N2	1.346 (4)	C19—H19B	0.9700
C7—C8	1.487 (4)	C20—O4	1.236 (4)
C8—C9	1.518 (4)	C20—O3	1.249 (4)
C8—H8A	0.9700	C20—Cd1^ii^	2.667 (4)
C8—H8B	0.9700	O3—Cd1^ii^	2.293 (2)
C9—C10	1.520 (4)	O4—Cd1^ii^	2.362 (2)
C9—H9A	0.9700	N1—Cd1^iii^	2.262 (3)
C9—H9B	0.9700	N2—H2A	0.8600
C10—O2	1.239 (4)	N4—H4A	0.8600
			
N1^i^—Cd1—N3	103.73 (10)	O2—C10—O1	123.4 (3)
N1^i^—Cd1—O1	106.08 (9)	O2—C10—C9	120.6 (3)
N3—Cd1—O1	93.38 (9)	O1—C10—C9	115.9 (3)
N1^i^—Cd1—O3^ii^	100.41 (9)	O2—C10—Cd1	70.45 (18)
N3—Cd1—O3^ii^	103.63 (10)	O1—C10—Cd1	52.94 (15)
O1—Cd1—O3^ii^	144.01 (9)	C9—C10—Cd1	168.8 (2)
N1^i^—Cd1—O4^ii^	146.52 (9)	N4—C11—C12	132.8 (3)
N3—Cd1—O4^ii^	104.51 (10)	N4—C11—C16	105.3 (3)
O1—Cd1—O4^ii^	89.85 (8)	C12—C11—C16	121.8 (3)
O3^ii^—Cd1—O4^ii^	55.44 (9)	C13—C12—C11	116.2 (4)
N1^i^—Cd1—O2	84.96 (8)	C13—C12—H12	121.9
N3—Cd1—O2	145.40 (8)	C11—C12—H12	121.9
O1—Cd1—O2	52.26 (7)	C12—C13—C14	122.5 (4)
O3^ii^—Cd1—O2	107.64 (9)	C12—C13—H13	118.8
O4^ii^—Cd1—O2	81.96 (8)	C14—C13—H13	118.8
N1^i^—Cd1—C20^ii^	124.62 (11)	C15—C14—C13	121.2 (4)
N3—Cd1—C20^ii^	106.84 (10)	C15—C14—H14	119.4
O1—Cd1—C20^ii^	116.79 (10)	C13—C14—H14	119.4
O3^ii^—Cd1—C20^ii^	27.87 (10)	C16—C15—C14	117.4 (4)
O4^ii^—Cd1—C20^ii^	27.60 (9)	C16—C15—H15	121.3
O2—Cd1—C20^ii^	94.44 (9)	C14—C15—H15	121.3
N1^i^—Cd1—C10	96.06 (9)	C15—C16—C11	120.9 (3)
N3—Cd1—C10	119.59 (10)	C15—C16—N3	130.4 (3)
O1—Cd1—C10	26.30 (8)	C11—C16—N3	108.6 (3)
O3^ii^—Cd1—C10	128.08 (10)	N3—C17—N4	111.4 (3)
O4^ii^—Cd1—C10	85.33 (9)	N3—C17—C18	125.4 (3)
O2—Cd1—C10	25.96 (8)	N4—C17—C18	123.3 (3)
C20^ii^—Cd1—C10	106.94 (11)	C17—C18—C19	114.6 (3)
C6—C1—C2	119.6 (3)	C17—C18—H18A	108.6
C6—C1—N1	109.1 (3)	C19—C18—H18A	108.6
C2—C1—N1	131.3 (3)	C17—C18—H18B	108.6
C3—C2—C1	118.2 (4)	C19—C18—H18B	108.6
C3—C2—H2	120.9	H18A—C18—H18B	107.6
C1—C2—H2	120.9	C18—C19—C20	114.4 (3)
C2—C3—C4	120.9 (4)	C18—C19—H19A	108.7
C2—C3—H3	119.5	C20—C19—H19A	108.7
C4—C3—H3	119.5	C18—C19—H19B	108.7
C5—C4—C3	121.7 (4)	C20—C19—H19B	108.7
C5—C4—H4	119.2	H19A—C19—H19B	107.6
C3—C4—H4	119.2	O4—C20—O3	121.3 (3)
C6—C5—C4	116.6 (4)	O4—C20—C19	119.9 (3)
C6—C5—H5	121.7	O3—C20—C19	118.8 (3)
C4—C5—H5	121.7	O4—C20—Cd1^ii^	62.30 (19)
C5—C6—N2	131.8 (4)	O3—C20—Cd1^ii^	59.13 (18)
C5—C6—C1	123.0 (4)	C19—C20—Cd1^ii^	176.2 (3)
N2—C6—C1	105.2 (3)	C10—O1—Cd1	100.76 (19)
N1—C7—N2	111.9 (3)	C10—O2—Cd1	83.59 (19)
N1—C7—C8	126.8 (3)	C20—O3—Cd1^ii^	93.0 (2)
N2—C7—C8	120.9 (3)	C20—O4—Cd1^ii^	90.1 (2)
C7—C8—C9	110.5 (3)	C7—N1—C1	105.5 (3)
C7—C8—H8A	109.5	C7—N1—Cd1^iii^	126.7 (2)
C9—C8—H8A	109.5	C1—N1—Cd1^iii^	127.2 (2)
C7—C8—H8B	109.5	C7—N2—C6	108.3 (3)
C9—C8—H8B	109.5	C7—N2—H2A	125.9
H8A—C8—H8B	108.1	C6—N2—H2A	125.9
C8—C9—C10	116.5 (3)	C17—N3—C16	106.3 (3)
C8—C9—H9A	108.2	C17—N3—Cd1	131.2 (2)
C10—C9—H9A	108.2	C16—N3—Cd1	121.4 (2)
C8—C9—H9B	108.2	C17—N4—C11	108.4 (3)
C10—C9—H9B	108.2	C17—N4—H4A	125.8
H9A—C9—H9B	107.3	C11—N4—H4A	125.8

Symmetry codes: (i) *x*, *y*−1, *z*; (ii) −*x*, −*y*, −*z*+1; (iii) *x*, *y*+1, *z*.

### Poly[bis[µ2-3-(1H-benzimidazol-2-yl)propionato]cadmium(II)] (2) Hydrogen-bond geometry (Å, º)

**Table:**

*D*—H···*A*	*D*—H	H···*A*	*D*···*A*	*D*—H···*A*
N2—H2*A*···O2^iv^	0.86	2.10	2.823 (4)	141
N4—H4*A*···O1^v^	0.86	2.03	2.862 (4)	161

Symmetry codes: (iv) −*x*+1, −*y*+1, −*z*+1; (v) −*x*, *y*−1/2, −*z*+1/2.
